# Cirurgia de Endocardite Infecciosa. Análise de 328 Pacientes Operados em um Hospital Universitário Terciário

**DOI:** 10.36660/abc.20220608

**Published:** 2023-03-01

**Authors:** Marcelo Serafim Jorge, Alfredo J. Rodrigues, Walter Vilella A. Vicente, Paulo Roberto B. Evora

**Affiliations:** 1 Departamento de Cirurgia e Anatomia Faculdade de Medicina de Ribeirão Preto Universidade de São Paulo São Paulo SP Brasil Departamento de Cirurgia e Anatomia – Faculdade de Medicina de Ribeirão Preto, Universidade de São Paulo, São Paulo, SP – Brasil

**Keywords:** Endocardite Infecciosa, Procedimentos Cirúrgicos Cardiovasculares, Prevalência, Comorbidade, Mortalidade

## Abstract

**Fundamento:**

A endocardite infecciosa (EI) refere-se à infecção da superfície endocárdica do coração e geralmente ocorre em valvas nativas ou protéticas.

**Objetivo:**

Este estudo teve como objetivo levantar dados de EI refletindo a terapêutica cirúrgica, em um Hospital Universitário do interior do estado de São Paulo – Brasil.

**Método:**

Abordagem retrospectiva e observacional de 328 pacientes com EI operados entre 1982 e 2020

**Resultados:**

Os principais dados (n=121/37%), insuficiência cardíaca congestiva (n=114/35%), valvopatia (n=92/28%), diabetes mellitus (n=85/26%), doença renal crônica (n=59/18%) e febre reumática (49/15%). A insuficiência renal é um dos principais e mais relevantes fatores de risco pré-cirúrgicos para um mau prognóstico.

**Conclusão:**

Para um melhor resultado clínico e cirúrgico é necessário o diagnóstico sindrômico e etiológico precoce da EI, principalmente em pacientes com múltiplas comorbidades.

**Figura Central:**
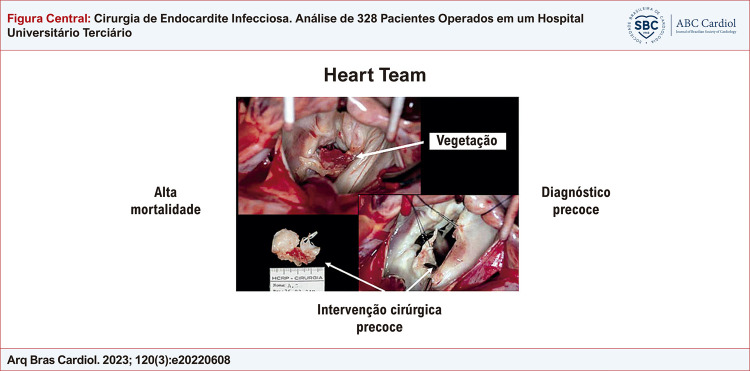
: Cirurgia de Endocardite Infecciosa. Análise de 328 Pacientes Operados em um Hospital Universitário Terciário

## Introdução

**Figura 1:**
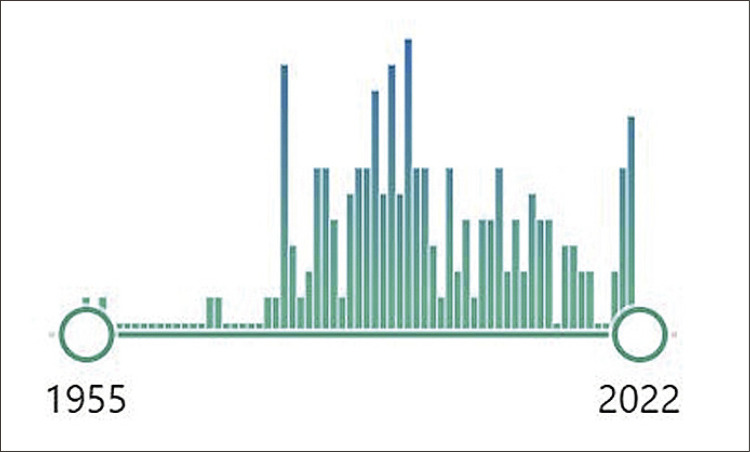
– Linha do tempo das publicações dos Arquivos Brasileiros de Cardiologia sobre endocardite infecciosa.

Conforme citado por Hubers et al.,^1^ Sir William Osler descreveu a endocardite infecciosa (EI) como “uma das mais formidáveis afecções cardíacas”, já que todos os pacientes naquela época morriam da doença. Embora a mortalidade tenha melhorado no último século, a EI continua sendo uma doença mortal, e mais avanços no diagnóstico e tratamento são necessários para continuar melhorando os resultados.^2^ Seria importante enfatizar sua importância epidemiológica com base em sua inclusão em metanálises. Por exemplo, Urina-Jassir et al.^3^ em excelente estudo, apresentam o perfil da endocardite na América Latina. Além disso, de 1955 a 2022, foram publicados 185 trabalhos indexados na MEDLINE (PUBMED) ( Figura 1 ). Há um “pico” de publicações nas décadas de 80 e 90, seguido de queda numérica. Tem-se a impressão de que um novo “pico” pode estar em andamento.

Acreditamos que estes breves dados sejam suficientes para definir o “racional” e a motivação para esta apresentação. Portanto, este estudo foi realizado para destacar a) A prevalência de comorbidades prévias dos pacientes; b) Indicação cirúrgica; c) Os principais fatores de risco que influenciam a mortalidade; e d) O prognóstico dos pacientes com EI submetidos à cirurgia como principal terapia.

## Pacientes e Métodos

### Delineamento de estudo

Realizou-se um estudo observacional retrospectivo. A amostra é caracterizada por pacientes com diagnóstico de EI operados, entre 1982 e 2020, no Hospital das Clínicas da Faculdade de Medicina de Ribeirão Preto da Universidade de São Paulo (HCFMRP-USP), sem distinção de sexo, idade, raça ou nível socioeconômico. A amostra populacional foi estabelecida por amostragem por conveniência.

### Aspectos éticos

A pesquisa está pautada em princípios éticos (Resolução nº 466, de 12 de dezembro de 2012, do Conselho Nacional de Saúde). O projeto foi aprovado pelo Comitê de Ética em Pesquisa do Hospital das Clínicas de Ribeirão Preto (HCRP), com registro na Plataforma CAAE Brasil: 32043720.9.0000.5440. Um termo assinado dispensou a necessidade de os pacientes assinarem o termo de consentimento livre e esclarecido.

### Instrumento de coleta de dados

Os pacientes foram classificados como EI pela codificação da alta hospitalar, pois as informações sobre a classificação de Duke não foram regularmente documentadas. Os pesquisadores tiveram acesso aos prontuários e, consequentemente, à lista de prontuários dos pacientes no Serviço de Arquivo Médico (SAM) da FMRP-USP no segundo semestre de 2020. Realizou-se uma criteriosa possível metanálise das informações relevantes de cada paciente que pudessem contribuir ao prognóstico e ao resultado cirúrgico. Esses dados foram considerados comorbidades, como hipertensão arterial sistêmica (HAS), insuficiência cardíaca (IC), doença coronariana (DC), fibrilação atrial (FA), diabetes, insuficiência renal e doenças pulmonares; abuso de álcool e/ou drogas ilícitas; tipo de válvula infectada e localização microrganismo causador; entre outros. Os dados foram coletados e tabulados para a devida análise e publicação do estudo.

### Análise de dados

Após a estruturação do banco de dados, realizou-se uma análise descritiva e exploratória. Para as variáveis categóricas, foram relatadas frequências e porcentagens. As variáveis contínuas foram apresentadas como média ± desvio padrão e as variáveis categóricas como porcentagens. Para análise de risco de mortalidade utilizou-se o cálculo de possibilidades ( *odds ratio* ) com intervalo de confiança de 95%. O teste do qui-quadrado foi utilizado para verificar as mudanças de prevalência. Os dados coletados foram analisados por meio do software Statistical Package for Social Science (SPSS, versão 20.0 [Inc. Chicago. IL]). Foram considerados valores significativos de p<0,05.

## Resultados

**Figura 2:**
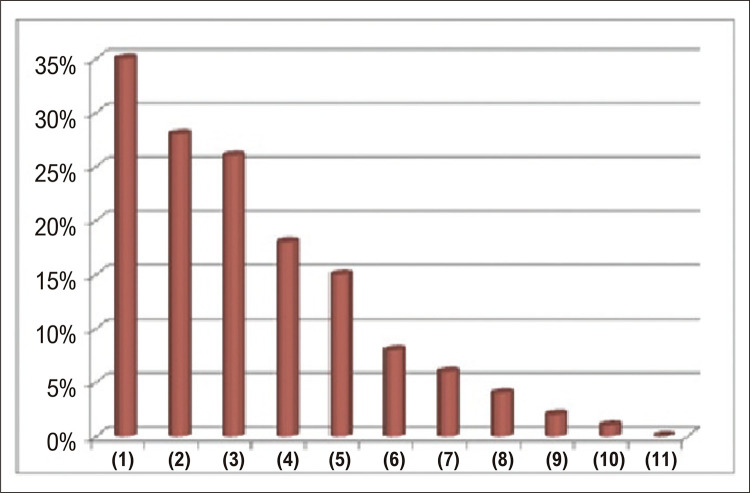
– Comorbidades prévias da amostra analisada (N=328). (1) Insuficiência cardíaca congestiva; (2) Doença valvar com prótese; (3) Diabetes Mellitus; (4) Doença renal crônica; (5) Febre reumática; (6) Endocardite prévia; (7) Imunocomprometido; (8) Hemodiálise; (9) Internação prolongada com cateter venoso central; (10) HIV positivo/AIDS (11) Toxicodependência intravenosa.

As cirurgias foram realizadas eletivamente em 88% dos pacientes devido à má resposta ao tratamento clínico e à imagem ecográfica. Os outros 12 pacientes foram operados em caráter de emergência por choque cardiocirculatório grave. No total, foram incluídos neste estudo 328 pacientes com EI operados de 1982 a 2020, com idade prevalente entre 41 e 60 anos. A Tabela 1 apresenta os dados demográficos dos pacientes, bem como os dados de mortalidade, classificação de EI e principais etiologias. As demais tabelas apresentam dados pré-operatórios, comorbidades prévias ( Figura 2 ), indicação cirúrgica ( Tabela 2 ), prognóstico ( Tabela 3 ) e principais riscos de mortalidade ( Tabela 4 ) de todos os pacientes operados.

**Tabela 1:** – Demografia e mortalidade (N=328)

1.Demografia	N=328
Homem	119 (36%)
Mulher	209 (64%)
**2. Mortalidade global**	160 (49%)
Mortalidade em homens	100 (63%)
Mortalidade em mulheres	60 (37%)
**3 Classificação**	**N=328**
Agudas e subagudas	308 (94%)
Valva inespecífica	20 (6%)
**4. Etiologia**	
Staphylococcus	121 (37%)
Streptococcus	105 (32%)
*Enterococcus* , gram-negativos e outras espécies de fungos	17 (5%)
Cultura negativa	85 (26%)
**5. Cirurgia**	**N=328**
Urgente	39 (12%)
Eletiva	289 (88%)
**6. Próteses valvares**	262 (80%)
Mecânicas	92 (35%)
Biológicas	170 (65%)
Valvuloplastia	66 (20%)

**Tabela 2:** – Indicação cirúrgica da amostra analisada (N=328)

Indicação cirúrgica	N	Freq.
Disfunção de Ejeção Cardíaca	230	70%
Falha no Tratamento Clínico	187	57%
Vegetação > 10mm	154	47%
Disfunção da válvula	102	31%
Embolia cardiogênica	39	12%

**Tabela 3:** – Prognóstico da amostra analisada (N=328)

Prognóstico	N	Freq.
Sobrevida	168	51%
Óbito	160	49%
Mortalidade Cirúrgica	8	2%

**Tabela 4:** – Principais Riscos de mortalidade (N=328)

Riscos de Mortalidade	Razão de chances (Odds ratio)	IC 95%	p
Idade	1,21	0,90 - 1,32	0,335
Insuficiência Renal	3,40	1,65 - 7,07	0,081
Insuficiência Cardíaca	0,91	0,72 - 1,66	0,090
Infecção Refratária ao Tratamento Clínico	2,35	1,65 - 5,40	0,001
Diabetes Mellitus	0,55	0,10 - 1,28	0,500
Embolismo	2,40	0,90 - 4,40	0,533

## Discussão

As principais comorbidades apresentadas pelos pacientes deste estudo foram: 1) Insuficiência cardíaca congestiva do ventrículo esquerdo (35%); 2) Valvulopatia com prótese (28%); 3) Diabetes Mellitus (26%); 4) Doença renal crônica (18%) e; 5) Febre reumática (15%) ( Figura 2 , Tabela 1 )). As principais indicações cirúrgicas foram: 1) Disfunção de ejeção do ventrículo esquerdo (n=230); decorrentes de ICC e/ou disfunções valvares; 2) Falha no tratamento clínico (n=187), e; 3) Vegetação ecocardiográfica maior que 10 mm (n=154).

Revisamos arquivos de 375 pacientes submetidos à cirurgia (69,3 óbitos; 30,7% sobreviventes), entre 1982 e 2020. Encontramos uma visão semelhante entre as referências mundiais, sugerindo uma espécie de “comportamento universal”. Essas observações reforçam o raciocínio da presente investigação. A cirurgia foi realizada eletivamente em 88% da amostra. Isso é muito incomum e deve ser especulado, pois a mortalidade é extremamente alta.

A análise epidemiológica revelou que o padrão etiológico da EI mudou nas últimas três décadas, quando o Staphylococcus se tornou mais prevalente (37%) do que o Streptococcus (32%).^4^ Alguns estudos sugerem que a infecção por S. aureus pode ser um importante preditor isolado de maior mortalidade em IE.^5^ Este evento é justificado pela associação deste germe com o crescimento de grandes vegetações valvares e abscessos, estando também relacionado a piores desfechos neurovasculares como acidentes vasculares.^6^Culturas negativas e, portanto, não identificação do patógeno ocorreu em 26 % de pacientes (n=85). Vários fatores podem contribuir para que as culturas diagnósticas sejam negativas, como técnicas microbiológicas inadequadas e uso prévio de antibioticoterapia empírica antes da análise diagnóstica, o que pode prejudicar o tratamento antimicrobiano guiado adequado, impactando o quadro clínico, desfecho e prognóstico do paciente.^7^

Para melhor estratificação de risco, cada caso deve ser individualizado, visando o melhor momento cirúrgico para o paciente. Ressalta-se que novas técnicas têm sido descritas, como a plastia da valva aórtica de Ozaki e a plastia da valva mitral transaórtica com pericárdio autólogo.^8^Na endocardite da valva tricúspide, de acordo com as diretrizes da European Society o Cardiology (ESC) e da American Association for Thoracic Surgery, o melhor reparo e preservação possível da válvula do paciente é a primeira escolha. Esses detalhes não foram considerados nesta apresentação. Não houve diferença na incidência geral de mortalidade (n=160/49%), sendo a mortalidade maior no sexo masculino (n=100/63%) do que no feminino (n=60/37%). A mortalidade neste estudo (49%) teve maior incidência em comparação com estudos recentes, que indicam taxas de mortalidade em torno de 8-21%.^9 , 10^ As próteses biológicas foram prevalentes em 170 (65%) pacientes; próteses mecânicas foram utilizadas em 92 (35%) pacientes e valvoplastias foram realizadas em 60 (20%) das operações. A preferência por próteses valvares mecânicas, levando em conta uma possível suscetibilidade à infecção, não é verdadeira. Não há diferenças entre próteses biológicas e mecânicas.

O reparo da válvula é preferível quando a anatomia o permite. O reparo da valva mitral é mais frequentemente realizado quando comparado ao reparo da valva aórtica, que raramente é bem-sucedido. No entanto, a substituição da valva aórtica por uma valva mecânica ou bioprotética é o manejo de escolha. Se o paciente tiver pressão e resistência pulmonar aumentada, não é aconselhável extirpar a válvula e deixar regurgitação grave. Em casos complexos com infecção não controlada localmente, a excisão total do tecido infectado e desvitalizado deve ser seguida de troca valvar. As diretrizes da European Society of Cardiology (ESC) e da American Association for Thoracic Surgery não favorecem as válvulas mecânicas ou bioprotéticas, pois apresentam mortalidade operatória semelhante. No entanto, quando há risco de sangramento pós-operatório ou transformação de lesões cerebrais em lesões hemorrágicas, as biopróteses valvares são preferíveis para evitar a anticoagulação do paciente que ao longo do período analisado pelo estudo varia de acordo com o tipo de agente infeccioso, a extensão do envolvimento do endocárdio, o grau de disfunção ventricular esquerda e a condição clínico-hemodinâmica antes da cirurgia.

A análise multivariada mostrou que existem dois principais fatores de risco para maior mortalidade com significância estatística, que são a Insuficiência Renal (OR 3,40; IC 95% 1,65 - 7,07; p=0,081); e a Infecção Refratária ao Tratamento Clínico (OR 2,35; IC 95% 1,65 - 5,40; p<0,001). Os demais fatores preditivos como Idade, Insuficiência Cardíaca, Diabetes Mellitus e Embolia não apresentaram relevância estatística na análise multivariada deste estudo ( Tabela 4 ).

Embora este estudo não seja completo, mostra uma clara necessidade de diagnóstico sindrômico e etiológico precoce da EI, principalmente em pacientes com múltiplas comorbidades e hospitalizados, para melhor resultado clínico e cirúrgico. As taxas de mortalidade são altas nesses pacientes, em parte devido à alta incidência de hemoculturas negativas^7^ e em parte pela dificuldade de abordagem cirúrgica em pacientes de alto risco cardiovascular e cirúrgico. O tratamento cirúrgico da EI é um desafio associado a significativa morbidade e mortalidade cirúrgica precoce, em pacientes com prognóstico clínico desfavorável, é uma oportunidade de aumentar a sobrevida do paciente, modificando o curso natural da doença.^8 - 10^

Ao final desta investigação, com os dados da revisão da literatura, fica clara a necessidade de um diagnóstico sindrômico e etiológico precoce da EI, principalmente em pacientes com múltiplas comorbidades e hospitalizados, para um melhor resultado clínico e cirúrgico. As taxas de mortalidade são altas nesses pacientes, em parte devido à alta incidência de hemoculturas negativas e em parte devido à dificuldade de abordagem cirúrgica de pacientes de alto risco cardiovascular e cirúrgico. Embora o tratamento cirúrgico da EI seja um desafio e associado a significativa morbidade e mortalidade, a intervenção cirúrgica precoce, em pacientes com prognóstico clínico ruim, deve ser uma oportunidade para aumentar a sobrevida do paciente, modificando o curso natural da doença. Portanto, o assunto é obviamente pertinente a um “Heart Team”, uma das razões da escolha dos Arquivos Brasileiros de Cardiologia para o presente relato. Ressaltamos, também, a contribuição para metanálises uma vez que estudos multicêntricos prospectivos são de elaboração prática quase impossível.

### Limitações do estudo

Por fim, é possível especular se as características do hospital envolvido no estudo (um hospital universitário terciário) podem ser responsabilizadas por certo viés de avaliação. Como regra geral, os pacientes são encaminhados para tratamento cirúrgico. Assim, considerações sobre as diretrizes atuais (tratamento com antibióticos, tempo para indicação cirúrgica, fatores de risco etc.) podem ser menos relevantes. Em outras palavras, o atendimento individualizado ao paciente deve ser considerado. Haveria necessidade científica de melhor separação de casos entre operadoras e tecnologia disponível no serviço, pois há um grande período entre 1982 e 2020. Essa avaliação foi pensada, mas a consulta retrospectiva dos prontuários não foi uniforme. Pode-se dizer que todos os casos foram operados por três professores, e as tecnologias de circulação extracorpórea e técnicas anestésicas já eram de boa qualidade. Como as observações, em geral, não apresentaram grandes diferenças com os dados disponíveis na literatura, é possível especular que esses dados não tenham influência relevante.
