# Supplementary material for: Cirurgia de Endocardite Infecciosa. Análise de 328 Pacientes Operados em um Hospital Universitário Terciário
Source: Arq Bras Cardiol. 2023 Mar 1;120(3):e20220608. [Article in Portuguese] doi: 10.36660/abc.20220608 (PMC12080617; doi:10.36660/abc.20220608)
Supplement: Translation

# Translation: Infective Endocarditis Surgery. Insights from 328 Patients Operated in a University Tertiary Hospital

**Authors:** Marcelo Serafim Jorge, Alfredo J. Rodrigues, Walter Vilella A. Vicente, Paulo Roberto B. Evora

**Affiliations:** 1 Brasil Departamento de Cirurgia e Anatomia – Faculdade de Medicina de Ribeirão Preto, Universidade de São Paulo, São Paulo, SP – Brasil

**Keywords:** Endocarditis, Non-Infective, Cardiovascular Surgical Procedures, Prevalence, Comorbidity, Mortality

## Abstract

**Background:**

Infectious endocarditis (IE) refers to infection of the endocardial surface of the heart and usually occurs in native or prosthetic valves.

**Objective:**

This study aimed to raise IE data reflecting the surgical therapy in a University Hospital in the interior of the State of Sao Paulo–Brazil.

**Method:**

Retrospective and observational approach of 328 patients with IE who underwent surgery between 1982 and 2020

**Results:**

The main data (n=121/37%), congestive heart failure (n=114/35%), valve disease (n=92/28%), diabetes mellitus (n=85/26%), chronic kidney disease (n=59/18%), and rheumatic fever (49/15%). Renal failure is one of the main and most relevant pre-surgical risk factors for a poor prognosis.

**Conclusion:**

For a better clinical and surgical outcome, an early syndromic and etiological diagnosis of IE is necessary, especially in patients with multiple comorbidities.

## Introduction

Hubers et al.^1^cited that Sir William Osler described IE as “one of the most formidable; of cardiac affections” since all patients in that era died of the disease. Although mortality has improved in the last century, IE continues to be a deadly disease, and further advancements in diagnosis and treatment are necessary to continue outcome improvements.^2^It would be important to emphasize its epidemiological importance based on its inclusion in meta-analyzes, as Urina-Jassir et al.^3^and his colleagues who, in an excellent study, present the profile of endocarditis in Latin America. In addition, from 1955 to 2022, 185 publications MEDLINE (PUBMED) indexed were published ( Figure 1 ). There is a “peak” of publications in the 80s and 90s, followed by a number drop. One has the impression that a new “peak” may be in progress.

**Figure 1:** – Time-course of MEDLINE publicações (infective endocarditis and cardiac surgery).

We guess these brief data are enough to define this presentation’s “rationale” and motivation. Therefore, this study was carried out to highlight a) The prevalence of previous comorbidities of patients; b) Surgical indication; c) The major risk factors that influence mortality; and d) The prognosis of patients with IE undergoing surgery as the main therapy.

## Patients and Methods

### Study design

I performed a retrospective observational study. The sample is characterized by patients diagnosed with IE who underwent surgery at the Hospital das Clínicas da Faculdade de Medicina de Ribeirão Preto da Universidade de São Paulo HCFMRP-USP, without distinction of sex, age, race, or socioeconomic status, between 1982 and 2020. The population sample was established through convenience sampling.

### Ethical aspects

This research is based on ethical principles (Resolution No. 466 of December 12, 2012, of the National Health Council). The research project was approved by the Research Ethics Committee of the Hospital das Clínicas de Ribeirão Preto (HCRP), with registration on the CAAE Platform Brazil: 32043720.9.0000.5440. A signed term dispensed with the need for patients to sign the free and informed consent form.

### Data collection instrument

The patients were classified as IE by hospital discharge coding because the information about the Duke classification was not useful. The researchers had access to medical records and, consequently, to the list of patient records in the Medical Archive Service (SAM) of FMRP in the second half of 2020. It was carried out a careful possible analysis of each patient’s relevant information that could contribute to the prognosis and surgical outcome. These data were namely comorbidities, such as systemic arterial hypertension (SAH), heart failure (HF), coronary disease (CD), atrial fibrillation (AF), diabetes, renal failure, and lung diseases; alcohol and/or illicit drug abuse; type of infected valve and location; causative microorganism; among others. Data were collected and tabulated for proper analysis and publication of the study.

### Data analysis

After structuring the database, they carried a descriptive and exploratory analysis out. For categorical variables, frequencies and percentages were reported. Continuous variables were presented as mean ± standard deviation, and categorical variables as percentages. For mortality risk analysis, odds ratios were calculated with a confidence interval of 95%. The chi-square test was used to verify changes in prevalence. The collected data were analyzed using the Statistical Package for Social Science software (SPSS, version 20.0 [Inc. Chicago. IL]). Significant values of p<0.05 were considered.

## Results

Surgery was done electively for 88% of patients based on poor response to the clinical treatment and echo image. The other 12 patients were operated on as emergency due to severe cardiocirculatory shock. In total, 328 patients with IE who underwent surgery from 1982 to 2020, with a prevalent age between 41 and 60 years, were included in this study. Table 1 presents the patients’ demographic data and the mortality, IE classification, and main etiologies data. The other tables present preoperative data, previous comorbidities ( Figure 2 ), surgical indication ( Table 2 ), prognosis ( Table 3 ), and main risks of mortality ( Table 4 ) of all operated patients.

**Table 1:** – Overview of the analyzed sample (N=328)

1. Demographic and mortality	N=328
Female	119 (36%)
Male	209 (64%)
**2. Global mortality incidence**	160 (49%)
Mortality in men	100 (63%)
Mortality in women	60 (37%)
**3. Classification**	**N=328**
Acute and subacute	308 (94%)
Non-specific valve	20 (6%)
**4. Etiology**	
*Staphylococcus* infection	121 (37%)
*Streptococcus* infection	105 (32%)
*Enterococcus* , gram-negative and }fungal species	17 (5%)
Negative culture	85 (26%)
**5. Surgery**	**N=328**
Emergency	39 (12%)
Elective	289 (88%)
**6. Prosthesis**	262 (80%)
Mechanical	92 (35%)
Biological	170 (65%)
Valvuloplasty	66 (20%)

**Figure 2:** – Previous comorbidities of the analyzed sample (n=328). (1) Congestive heart failure; (2) Valve disease with prosthesis; (3) Diabetes mellitus; (4) Chronic kidney disease; (5) Rheumatic fever ;(6) Previous endocarditis; (7) Immunocompromised; (8)Hemodialysis; (9)Prolonged hospitalization with a central venous catheter; (10) HIV positive/AIDS; (11) Intravenous drug addiction.

**Table 2:** – Surgical indication of the analyzed sample (N=328)

Surgical Indication	N	Freq.
Cardiac Ejection Dysfunction	230	70%
Clinical Treatment Failure	187	57%
Vegetation >10mm	154	47%
Valve Dysfunction	102	31%
Cardiogenic Embolism	39	12%

**Table 3:** – Prognosis of the analyzed sample (N=328)

Prognosis	N	Percentage
Survival	168	51%
Death	160	49%
Surgical mortality	8	2%

**Table 4:** – Analysis of the main mortality risks (N=328)

Mortality Risks	OR	CI – 95 95%	p
Age	1.21	0.90 - 1.32	0.335
Renal insufficiency	3.40	1.65 - 7.07	0.081
Cardiac insufficiency	0.91	0.72 - 1.66	0.090
Infection Refractory to Clinical Treatment	2.35	1.65 - 5.40	0.001
Diabetes Mellitus	0.55	0.10 - 1.28	0.500
Embolism	2.40	0.90 - 4.40	0.533

*OR: odds rate; CI: confidence interval; p: significance.*

## Discussion

The main comorbidities presented by patients in this study were: 1) Left ventricle congestive heart failure (35%); 2) Valve disease with a prosthesis (28%); 3) Diabetes Mellitus (26%); 4) Chronic kidney disease (18%), and; 5) Rheumatic fever (15%) ( Figure 2 , Table 1 )). The main surgical indications were: 1) Left ventricular ejection dysfunction (n=230); resulting from CHF and/or valvular dysfunctions; 2) Failure of clinical treatment (n=187), and; 3) Echocardiography vegetation larger than 10 mm (n=154).

We reviewed files of 375 under-surgery patients (69.3 of death; 30.7% survivors) between 1982 and 2O2O. We found a similar overview among worldwide references, suggesting a “universal behavior.” These observations reinforce the rationale of the present investigation. Surgery was done electively for 88% of the sample. This is most unusual and should be speculated as the reason mortality is exceedingly high.

The epidemiological analysis revealed that the etiological pattern of IE had changed over the last three decades when *Staphylococcus* became more prevalent (37%) than *Streptococcus* (32%).^4^Some studies suggest that *S. aureus* infection may be an important isolated predictor of higher mortality in IE.^5^This event is justified by the association of this germ with the growth of large valve vegetations and abscesses, also related to worse neurovascular outcomes such as cerebrovascular accidents.^6^Negative cultures and no identification of the pathogen occurred in 26% of patients (n=85). Several factors can corroborate for the diagnostic cultures to be negative, such as inadequate microbiological techniques and previous use of empirical antibiotic therapy before the diagnostic analysis, which can impair the appropriate guided antimicrobial treatment, affecting the clinical outcome and prognosis of the patient.^7^

Each case must be individualized for better risk stratification, aiming at the best surgical moment for the patient. It needs to emphasize that new techniques have been described, such as Ozaki aortic valve repair and transaortic mitral valve repair using autologous pericardium.^8^In tricuspid valve endocarditis, per both the European Society o Cardiology (ESC) and American Association for Thoracic Surgery guidelines, the best possible repair, and preservation of the patient’s valve is the first choice. These details were not considered in this presentation. There was no difference in the overall incidence of mortality (n=160/49%), with mortality being higher in males (n=100/63%) than in females (n=60/37%). Mortality in this study (49%) was higher than in recent studies, indicating mortality rates of around 8-21%.^9 , 10^Biological prostheses were prevalent in 170 (65%) patients; mechanical prostheses were used in 92 (35%) patients, and valvuloplasties were performed in 60 (20%) of the operations. The preference for mechanical valve prostheses, considering a possible susceptibility to infection, is not true. There are no differences between biological and mechanical prostheses.

Valve repair is preferable when the anatomy allows it. Mitral valve repair is more frequently achievable when compared with aortic valve repair, which is seldom successful. Nevertheless, aortic valve replacement with a mechanical or bioprosthetic valve is the management of choice. If the patient has increased pulmonary pressure and resistance, excising the valve and leaving severe regurgitation are not advisable. In complex cases with locally uncontrolled infection, total excision of infected and devitalized tissue should be followed by valve replacement. European Society of Cardiology (ESC) and the American Association for Thoracic Surgery guidelines do not favor mechanical or bioprosthetic valves, as they have similar operative mortality. However, when there is a risk for postoperative bleeding or transformation of brain lesions into hemorrhagic lesions, bioprosthetic valves are preferable to avoid anticoagulation. The patient’s prognosis, considering death due to IE complications, survival, and surgical mortality as the only three possible outcomes for the patient over the period analyzed by the study, varies according to the type of infectious agent, the extent of involvement of the endocardium, the degree of left ventricular dysfunction and the clinical-hemodynamic condition of the patient before surgery.

Multivariate analysis showed that there were two main risk factors for higher mortality with statistical significance, which are Renal Failure (OR 3.40; 95% CI 1.65 - 7.07; p=0.081); and Infection Refractory to Clinical Treatment (OR 2.35; 95% CI 1.65 - 5.40; p<0.001). The other predictive factors, such as Age, Heart Failure, Diabetes Mellitus, and Embolism, did not show statistical relevance in the multivariate analysis in this study ( Table 4 ).

At the end of this investigation, with the literature review data, there is an apparent need for an early syndromic and etiological diagnosis of IE, especially in patients with multiple comorbidities and hospitalized, for a better clinical and surgical outcome. Mortality rates are high in these patients, partly due to a high incidence of negative blood cultures^7^and partly due to difficulty surgically approaching patients at high cardiovascular and surgical risk. However, surgical treatment of IE is a challenge and is associated with significant morbidity and mortality. Early surgical intervention in patients with a poor clinical prognosis should be an opportunity to increase patient survival, modifying the natural course of the disease.^8 - 10^The subject is obviously a “Heart Team,” one of the reasons for choosing the *Arquivos Brasileiros de Cardiologia* for the present report ( Central Illustration ). We also emphasize the contribution of meta-analyses since multicenter prospective studies are practically impossible to prepare.

**Central Illustration:** : Infective Endocarditis Surgery. Insights from 328 Patients Operated in a University Tertiary Hospital

### Study limitations

Finally, it is possible to speculate whether the hospital characteristics involved in the study (a tertiary teaching hospital) can be blamed for a certain evaluation bias. As a general rule, patients are referred for surgical treatment. Thus, considerations about current guidelines (treatment with antibiotics, time to surgical indication, risk factors, etc.) may be less relevant. In other words, individualized patient care must be considered. There would be a scientific need for better separation of cases between operators, and technology available in the service, as there is a considerable period between 1982 and 2022. This evaluation was considered, but the retrospective consultation of medical records was not uniform. It can be said that three professors operated on all cases, and the technologies of extracorporeal circulation and anesthetic techniques were already of good quality. As the observations generally did not show great differences with the data available in the literature, it is possible to speculate that these data do not have a relevant influence.
